# Prolonged treatment with vitamin D in postmenopausal women with primary hyperparathyroidism

**DOI:** 10.1530/EC-12-0008

**Published:** 2012-06-08

**Authors:** Ranganathan R Rao, Harpal S Randeva, Sailesh Sankaranarayanan, Murthy Narashima, Matthias Möhlig, Hisham Mehanna, Martin O Weickert

**Affiliations:** 1 Warwickshire Institute for the Study of Diabetes, Endocrinology and Metabolism University Hospitals Coventry and Warwickshire NHS Trust Coventry UK; 2 Warwick Medical School, Clinical Sciences Research Institute University of Warwick Coventry UK; 3 Department of Endocrinology, Diabetes and Nutrition Charité-University-Medicine Berlin Berlin Germany; 4 Institute of Head and Neck Studies and Education (InHANSE) University Hospitals Coventry and Warwickshire NHS Trust Coventry UK

**Keywords:** parathyroids, hormone action, calcium, bone

## Abstract

**Introduction/background:**

Vitamin D deficiency further increases circulating parathyroid hormone (PTH) levels in patients with primary hyperparathyroidism (pHPT), with potential detrimental effects on bone mass.

**Methods:**

This was an observational clinical study in consecutive conservatively treated postmenopausal women (*n*=40) with pHPT and coexistent 25-hydroxyvitamin D deficiency (25OHD ≤50 nmol/l (≤20 ng/ml)). Patients who showed an increase in serum 25OHD above the threshold of vitamin D deficiency (>50 nmol/l; *n*=28) using treatment with various commonly prescribed vitamin D preparations were, for the purposes of statistical analyses, allocated to the treatment group. Patients who were retrospectively identified as having received no treatment with vitamin D and/or remained vitamin D deficient were considered as non-responders/controls (*n*=12). Adjusted calcium (adjCa), PTH and 25OHD concentrations were monitored in all subjects up to 54 months (mean observation period of 18±2 months).

**Results:**

Prolonged increased vitamin D intake, regardless of the source (serum 25OHD, increase from 32.2±1.7 nmol/l at baseline to 136.4±11.6 nmol/l, *P*<0.0001), significantly reduced serum PTH (13.3±1.1 vs 10.5±1.0 pmol/l, *P*=0.0001), with no adverse effects on adjCa levels (2.60±0.03 vs 2.60±0.02 mmol/l, *P*=0.77) and renal function tests (*P*>0.73). In contrast, serum PTH remained unchanged (15.8±2.6 vs 16.3±1.9 pmol/l, *P*=0.64) in patients who remained vitamin D deficient, with a significant difference between groups in changes of PTH (*P*=0.0003). Intrapartial correlation analyses showed an independent negative correlation of changes in 25OHD with PTH levels (*r*_ic_=−0.41, *P*=0.014).

**Conclusions:**

Prolonged treatment with vitamin D in various commonly prescribed preparations appeared to be safe and significantly reduced PTH levels by 21%.

## Introduction

Primary hyperparathyroidism (pHPT) in postmenopausal women is frequently associated with vitamin D deficiency (1, 2), which in turn is an additional stimulus for further increases in circulating parathyroid hormone (PTH) levels (3, 4, 5). Recent studies indicate that treatment of vitamin D deficiency may have the potential to reduce circulating PTH levels in patients with pHPT, without adverse effects on serum-adjusted calcium (adjCa) levels (3, 4, 5). Data regarding the effects of treatment with vitamin D in patients with pHPT are sparse. However, in the general population, beneficial effects of vitamin D replacement on bone mass and risk of fractures have been reported (6), as well as various additional factors that could argue for replacement with vitamin D. These include non-skeletal effects such as improved neuromuscular function and protection from hypertension, heart failure and resistance to infection (7). Furthermore, vitamin D deficiency may worsen cognition and lower mood in the elderly (8, 9), symptoms that are commonly associated with pHPT. Finally, there is an increased risk of severe and potentially life-threatening hypocalcaemia in patients with combined pHPT and vitamin D deficiency following parathyroidectomy (10), whereas correcting vitamin D deficiency has been shown to reduce both the incidence of post-operative hypocalcaemia and the length of stay in hospital (11).

More recent guidelines encourage vitamin D replacement to serum 25-hydroxyvitamin D (25OHD) levels above 50 nmol/l in all monitored patients with pHPT (5). However, only a few years ago recommendations regarding careful (400–600 IU/day) replacement of vitamin D in these patients even in the short-term were vaguer, pointing out that in some patients serum calcium levels could rise further with this treatment (12). Consequently, not all physicians have routinely replaced vitamin D deficiency in all patients with pHPT (5, 12, 13, 14, 15). We exploited this fact for the purpose of the present analysis, including both patients who had received prolonged treatment with vitamin D (both prospectively and retrospectively) and patients who were not treated with vitamin D for a comparable period (retrospectively only).

In clinical practice, a variety of vitamin D preparations are available, which include vitamin D (colecalciferol and ergocalciferol), combined vitamin D/calcium preparations, and/or dietary advice (vitamin D-rich foods, fish oil preparations, and exposure to sunlight). However, it is not known whether these agents are effective and safe in reducing PTH levels in postmenopausal women with pHPT and concomitant vitamin D deficiency, particularly in the longer term. In this study, we explored the effects of prolonged treatment with vitamin D on changes in circulating PTH and adjCa levels in postmenopausal women with pHPT and coexistent vitamin D deficiency.

## Materials and methods

### Patients

This was an observational clinical study. The outcome measures of this analysis were whether prolonged treatment with vitamin D in commonly used preparations is effective and results in relevant changes in circulating PTH and serum adjCa levels. Research was limited to secondary use of information previously collected in the course of normal care and data were anonymised before the conduction of statistical analyses. Therefore, this research did not fulfil the requirements for Research Ethics Committee (REC) review, in accordance with the Governance Arrangements for Research Ethics Committees (GAfREC), published by the UK Health Department in May 2011 (http://nres.nhs.uk/applications/approval-requirements/ethical-review-requirements). All clinical investigations were conducted in accordance with the guidelines in the Declaration of Helsinki.

**Figure 1:**
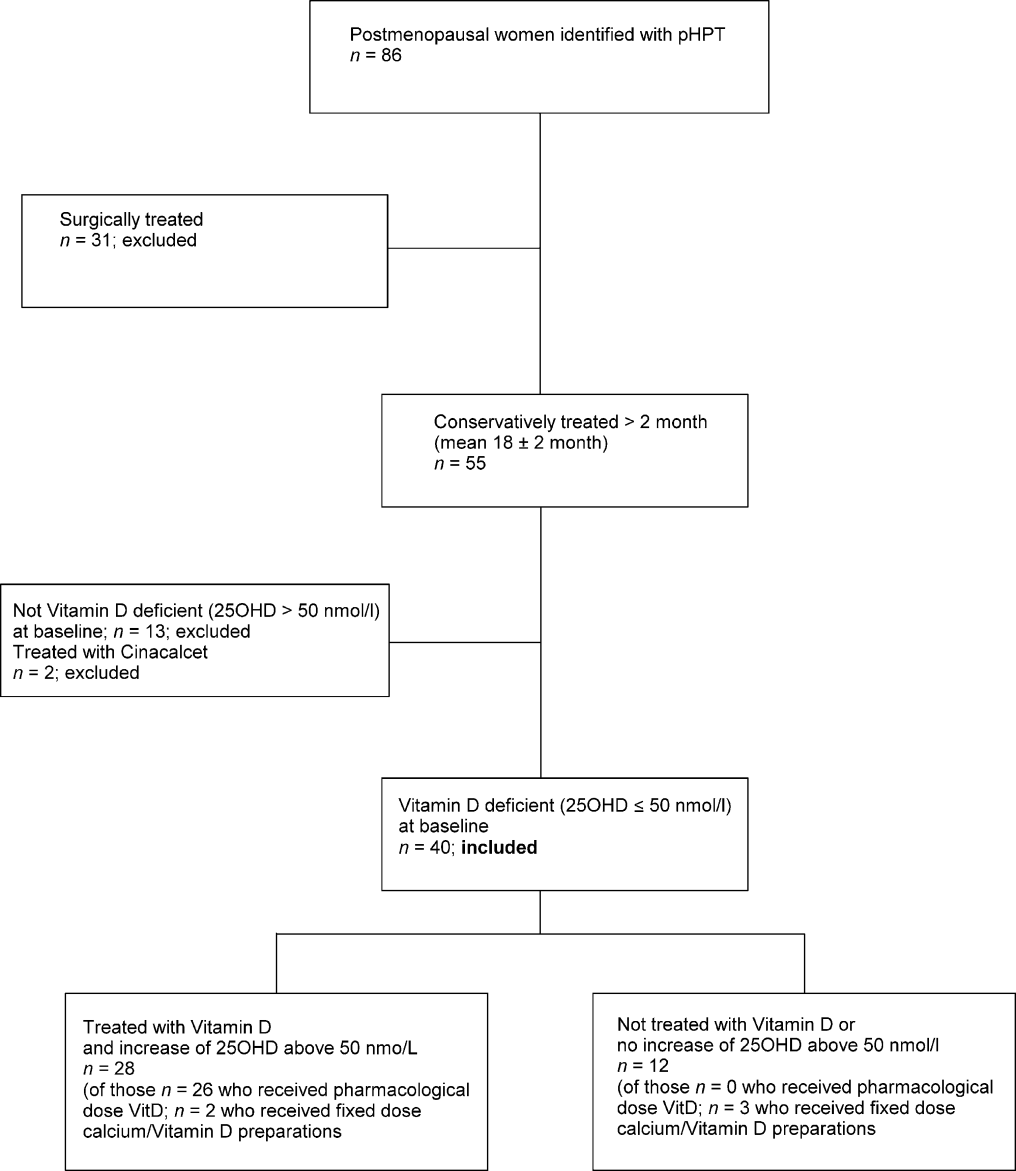
Flow of patients throughout the study. We identified 86 postmenopausal women who presented with pHPT in the University Hospital between 2003 and 2011. The flow of patients throughout the study is shown in the figure

A diagnosis of pHPT was made if both adjCa and PTH levels were above the upper limit of normal of assays used here (adjCa >2.58 mmol/l and PTH >4.2 pmol/l). Vitamin D deficiency was defined as 25OHD ≤50 nmol/l (≤20 ng/dl) and vitamin D insufficiency as 25OHD ≤75 nmol/l (≤30 ng/dl) (4, 16). We identified all postmenopausal women with pHPT and coexistent vitamin D deficiency who i) had attended our Endocrine Outpatient Clinics or Joint Thyroid Clinics in University Hospitals Coventry and Warwickshire NHS Trust from 2003 to 2011, ii) who were, due to comorbidities or risk of anaesthesia, not eligible for or currently not willing to have parathyroid surgery performed, and iii) who had at least two complete sets of paired measurements for adjCa, 25OHD and PTH levels available during the respective treatment/observation period. Figure 1 shows the flow of patients in this study.

Only patients who received prolonged (>2 month; mean 18±2 months) conservative treatment for pHPT were included, or patients who were retrospectively identified as not having received treatment with vitamin D for a comparable observation period.

Data from 40 consecutive postmenopausal women (age 69±2 years) with pHPT and coexistent vitamin D deficiency and fulfilling all the above criteria were extracted from the electronic clinical hospital database.

### Vitamin D replacement

In agreement with the recent suggestions (5, 17), vitamin D-deficient patients with pHPT who are seen in the Endocrine Outpatient Clinics of the corresponding author and who show adjCa levels below 3 mmol/l at baseline (18) are encouraged to increase the intake of vitamin D from natural sources and receive additional replacement with colecalciferol tablets 40 000–50 000 units (1000–1250 μg) per month, dependent on the availability of the respective preparation in the hospital pharmacy. Doses are adapted/reduced according to biochemical response. In none of the patients were dose adjustments necessary because of increases in hypercalcaemia, but dose was reduced in four patients who showed increases in serum 25OHD above 200 nmol/l. For clinical reasons (e.g. potential worsening of hypercalcaemia regardless of treatment with vitamin D; potential natural disease progression), all patients are prospectively observed with close monitoring of clinical symptoms and routinely performed blood tests. Patients who were seen in other clinics (*n*=29) were retrospectively identified. These patients were either treated with various vitamin D preparations (colecalciferol 20 000 units (500 μg), two tablets per month for up to 12 month; colecalciferol 50 000 units (1250 μg), one tablet per month for up to 12 month, up to colecalciferol 50 000 units (1250 μg) 1 tablet per week for up to 8 weeks; and i.m. injections of ergocalciferol, up to 300 000 units (7500 μg) as a single dose) and/or natural sources of vitamin D such as exposure to sun light, increased consumption of oily sea fish, e.g. salmon, mackerel and sardines, or fish oil supplements and/or no treatment with vitamin D was prescribed. Five of the patients were identified as having received a combination of calcium and vitamin D replacement preparation (containing colecalciferol 400 units (10 μg) and calcium carbonate 2.5 g daily), with three of them remaining vitamin D deficient during the observation period. This may be related to the relative small dose of vitamin D provided and/or to problems with long-term adherence with this medication. All patients were closely monitored for potential changes in adjCa levels.

For the purposes of analyses, the subjects were divided into two groups: a group of 28 patients who were treated with vitamin D and who showed increases in serum 25OHD above the threshold of vitamin D deficiency (>50 nmol/l) and a non-responder/control group of 12 patients who were retrospectively identified as having received no or insufficient treatment with vitamin D, defined as serum 25OHD levels remaining ≤50 nmol/l during the observation period. The observation period was comparable in patients who received treatment with vitamin D and patients who did not receive treatment with vitamin D and/or remained vitamin D deficient (17±3 vs 18±4 months, *P*=0.78). The observation period was more than 2 years in 11 patients, and a further ten patients were observed for more than 1 year. One patient was observed for 2 months, one patient for 3 months and the remaining 17 patients were observed for between 4 and 10 months.

Twenty of the patients (15 patients in the vitamin D treatment group and five patients in the non-responder/control group) were treated with bisphosphonates. Patients who received treatment with cinacalcet were not included in the analyses. None of the patients were treated with thiazide diuretics or other agents that are known to increase serum adjCa levels. Clinical details of all subjects, including duration of treatment with vitamin D are summarized in Table 1.

### Measurements

All measurements were performed using routine methods in the Pathology Department, University Hospitals Coventry and Warwickshire, UK. Serum 25OHD and PTH levels were measured using Roche Modular Analytics E170 (Roche Diagnostics GmbH) and alkaline phosphatase (ALP) and adjCa levels were analysed using Roche Modular Analytics P Module (Roche Diagnostics GmbH). The normal ranges of the assays were as follows: PTH, 1.1–4.2 pmol/l; adjCa, 2.10–2.58 mmol/l; and ALP, 35–105 U/l.

### Statistical analysis

Data are expressed as mean±s.e.m. Normal distribution was investigated using Kolmogorov–Smirnov test. Data are given both as absolute values and after correction for the baseline (day 1 of the observation period). One-way ANOVA was used for the detection of differences between vitamin D-treated and non-responders/controls. *T*-tests for independent samples were used for subgroup analyses. Two-tailed Student's *t*-test was used for longitudinal comparisons within groups. Pearson correlation coefficients were used to examine the relationships between variables. Intrapartial correlation analyses were additionally performed to detect independent relationships between variables. Stepwise regression analysis was performed to investigate the potential influence of changes in serum 25OHD and adjCa, as well as effects of age, body mass index (BMI) and use of bisphosphonates on changes in PTH levels. Further details are given in the respective parts of the Results section. *P*<0.05 was considered statistically significant. Analyses were performed using SPSS version 16 (Chicago, IL, USA).

## Results

**Figure 2:**
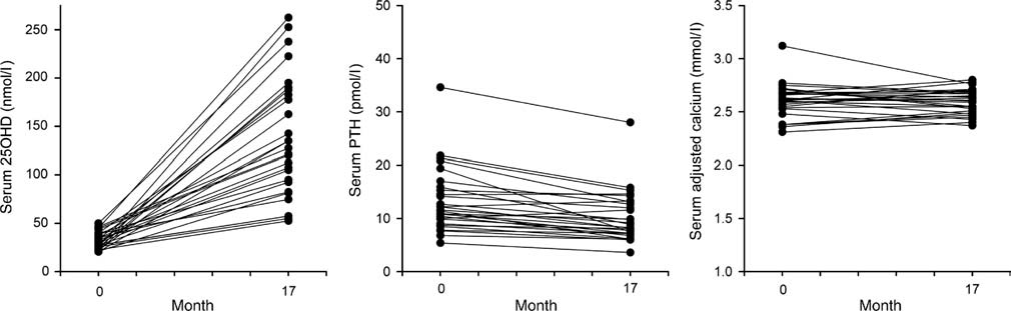
Changes in 25OHD, PTH and adjCa levels in *n*=28 postmenopausal women with pHPT and coexistent hypovitaminosis D who were treated with vitamin D in various preparations and doses (colecalciferol, ergocalciferol and/or natural sources) and showed an increase in serum 25OHD levels above the threshold of vitamin D deficiency (>50 nmol/l) during a mean observation period of 17±3 months. To convert serum 25OHD values to nanograms per millilitre, divide by 2.5.

**Figure 3:**
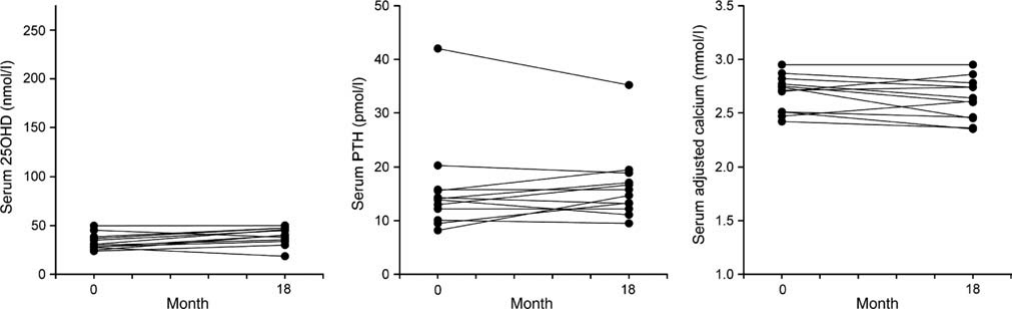
Changes in 25OHD, PTH and adjCa levels in *n*=12 postmenopausal women with pHPT and coexistent hypovitaminosis D who were not treated with vitamin D and/or showed no increase in serum 25OHD levels above 50 nmol/l during a mean observation period of 18±4 months. To convert serum 25OHD values to nanograms per millilitre, divide by 2.5.

The characteristics of the participants at baseline (day 1 of the observation period) are summarised in Table 1. Changes in absolute concentrations of serum 25OHD, PTH and adjCa after a mean observation period of 18±2 months are shown in the Figs 2 and 3.

Circulating PTH concentrations were comparable between groups at baseline (Table 1) but significantly different between the vitamin D-deficient and the non-vitamin D-deficient groups after the observation period, both for absolute concentrations of PTH (ANOVA, *P*=0.007) and for changes of PTH relative to the baseline (ANOVA, *P*=0.0003). As could be expected, changes in 25OHD were also different between groups (ANOVA, *P*<0.0001), where there were no differences in adjCa and ALP levels (ANOVA, *P*=0.51 and *P*=0.22 respectively).

Changes in 25OHD, PTH and adjCa levels in the group of 28 patients that were treated with vitamin D over a mean period of 17±3 months and showed an increase in serum 25OHD of at least above 50 nmol/l are shown in Fig. 2. This group included two of the patients that were treated with combined calcium/vitamin D preparations. The observed increase in serum 25OHD (from 32.2±1.7 to 136.4±11.6 nmol/l, *P*<0.0001) was associated with significantly reduced levels of both serum PTH (13.3±1.1 vs 10.5±1.0 pmol/l, *P*=0.0001) and ALP (79.4±6.7 vs 68.9±5.8 U/l, *P*=0.017). No adverse effects on serum adjCa levels were observed (2.60±0.03 vs 2.60±0.02 mmol/l, *P*=0.77). Circulating PTH levels decreased in 89% (25/28) of the patients who were treated with vitamin D, with a mean decrease in circulating PTH of 21% (Fig. 2).

Changes in 25OHD, PTH and adjCa levels in the group of 12 patients who were not or insufficiently treated with vitamin D and remained vitamin D deficient (control group for the purposes of statistical analyses) during a mean observation period of 18±4 months are shown in Fig. 3. This group included three of the patients who were treated with combined calcium/vitamin D preparations, but who showed no increase in serum 25OHD levels above the threshold of vitamin D deficiency. In untreated or insufficiently vitamin D replaced patients, serum PTH remained unchanged (15.8±2.7 vs 16.3±1.9 pmol/l, *P*=0.64) and significantly increased by 38% (from 12.3±2.2 to 17.0±1.4 pmol/l, *P*=0.035) in the subset of patients who did not show any increase or showed a further reduction of serum 25OHD during the observation period. In contrast to the changes observed in the group of patients who received treatment with vitamin D, in the 12 patients that did not receive treatment with vitamin D and/or whose 25OHD levels remained below 50 nmol/l, circulating PTH levels remained unchanged or even increased in 58% (7/12) of the patients, with a mean rise in PTH of 8% (Fig. 3).

No adverse effects on calcium levels were seen in any of the patients who were treated with vitamin D, irrespective of the type of vitamin D replacement and including the five patients who were treated with combined calcium/vitamin D preparations (baseline 2.59±0.09 vs 2.53±0.07 mmol/l after the observation period, *P*=0.37). However, as could be expected, the prescribed dose of vitamin D in combined calcium/vitamin D preparations was insufficient to increase serum 25OHD levels above the threshold in three of the five patients (Fig. 3).

Mean urinary calcium excretion, mainly measured at baseline before treatment with vitamin D was started, was 7.0±0.8 mmol/collection (reference range 2.5–6.2 mmol/collection). None of the patients developed symptomatic renal stones during the observation period of 18±2 months. Baseline levels for serum creatinine and estimated glomerular filtration rate (eGFR) are summarized in Table 1. At the end of the observation period, renal function tests remained unchanged in both vitamin D-treated (serum creatinine 69±3 μmol/l, *P*=0.73 vs baseline levels; and eGFR 85±5 ml/min per 1.73 m^2^, *P*=0.90 vs baseline levels) and untreated/insufficiently treated patients (serum creatinine 84±8 μmol/l, *P*=0.31, vs baseline levels; and eGFR 68±7 ml/min per 1.73 m^2^, *P*=0.21, vs baseline levels), with differences between groups in serum creatinine (*P*=0.001) but not in eGFR (*P*=0.075) reflecting the observed differences between groups at baseline (Table 1). None of the patients developed clinical fractures during the observation period.

Bivariate correlation of changes in absolute concentrations of 25OHD, PTH, adjCa and ALP levels showed a highly significant correlation of adjCa with PTH levels only (*r*=0.50, *P*=0.001). However, after correction for baseline levels, this correlation disappeared (*r*=−0.22, *P*=0.17), and a significant negative correlation between changes of 25OHD with PTH levels was detected (*r*=−0.49, *P*=0.001), as well as a correlation between changes of PTH with ALP levels (*r*=0.34, *P*=0.04). Intrapartial correlation analysis with correction for changes in adjCa and ALP levels confirmed a significant negative correlation of changes in 25OHD with PTH levels (*r*_*ic*_=−0.41, *P*=0.014), indicating that the magnitude of the increase in 25OHD was related to the observed decrease in circulating PTH levels. Otherwise, when adjusting for changes in PTH and adjCa levels, the observed correlation between changes in 25OHD and ALP disappeared (*r*_*ic*_=0.26, *P*=0.13). Stepwise regression analyses including changes in 25OHD and adjCa levels, age, BMI and use of bisphosphonates identified 25OHD (*P*<0.0002) and BMI (*P*=0.013) as predictors for changes in circulating PTH (adjusted *R* ^2^=0.36), but neither use of bisphosphonates (*P*=0.99) nor changes in serum adjCa levels (*P*=0.56) and age (*P*=0.11) showed an effect on this outcome.

Twenty-four of the patients who were treated with vitamin D showed an increase in serum 25OHD above 75 nmol/l (>30 ng/ml), which is commonly used as the threshold for vitamin D insufficiency (19). In these patients, the PTH-lowering effect of treatment with vitamin D was comparable with the changes seen in the vitamin D-deficient group: an increase in serum 25OHD from 33.2±1.8 to 149.9±11.4 nmol/l (*P*<0.0001) resulted in a 22% significant reduction of serum PTH levels (13.7±1.3 vs 10.7±1.2 pmol/l, *P*=0.0003), again with no adverse effects on adjCa (2.60±0.03 vs 2.59±0.02 mmol/l, *P*=0.68).

## Discussion

The prevalence of vitamin D deficiency is high in postmenopausal women with pHPT (20), with potential detrimental effects on bone mass (6). Although prescription of vitamin D is likely to be safe in patients with mild pHPT (3, 21, 22), at present it is not clear whether routine vitamin D supplementation should be offered as part of long-term medical treatment (17). Consequently, recent guidelines mention research on vitamin D replacement in pHPT as one of the blueprint areas for future research (5).

In this study, we show that prolonged treatment of postmenopausal women with pHPT and coexisting vitamin D deficiency for up to 54 months with various vitamin D preparations significantly reduced serum PTH concentrations, with no adverse effects on serum adjCa levels and renal function tests. Our results of decreasing PTH with vitamin D treatment are consistent with reports from other cohorts of patients with pHPT which include epidemiological observations (15, 23, 24), a large prospective observation reporting effects of high-dose preoperative vitamin D replacement therapy for 28 days (25), a 10-week prospective audit in 56 patients (4) and a 12-month prospective audit in 21 patients (3).

In the only available longer term audit so far investigating the effects of treatment with vitamin D in pHPT patients, relative high doses of colecalciferol were used to treat vitamin D deficiency (50 000 units (1250 μg), one tablet per week for 1 month, followed by 50 000 units (1250 μg), one tablet per month for 1 year) (3). In our study, using smaller doses in most of the patients, significant reductions in circulating PTH levels were observed. Some of the patients showed elevations of 25OHD above 250 nmol/l, although they remained below the toxic range of above 374 nmol/l (26). In fact, two of the patients that showed the highest increases in serum 25OHD had received lower doses of vitamin D replacement (800 units (20 μg) per day and 50 000 units (1250 μg) per month respectively) compared with the study of Reid *et al*. (3), indicating that increases in serum levels of 25OHD cannot be reliably predicted in all patients and should be monitored.

Various factors may argue for the replacement of vitamin D deficiency in patients with pHPT: suboptimal dietary intake of vitamin D is known to stimulate parathyroid adenoma growth unrelated to hypocalcaemia and active vitamin D (1-25OH_2_D) levels and reduces the calcaemic response to PTH (14). Apart from the elevated markers of increased bone turnover (27, 28), patients with coexisting pHPT and vitamin D deficiency are more likely to have a larger parathyroid adenoma and higher PTH levels (10, 14). Constantly elevated levels of PTH *per se* have detrimental effects on bone mass density and bone structure (29), primarily due to up-regulation of nuclear factor κB ligand (RANKL) and inhibition of osteoprotegerin expression, leading to an increase in osteoclast formation and activity (30, 31). Therefore, combined pHPT and vitamin D deficiency may introduce additional (secondary) stimulation to the parathyroid glands (10, 14), with further negative effects on bone mass. Importantly, even moderate restriction of vitamin D intake has been shown to increase PTH secretion, without reducing adjCa levels, whereas bone turnover is increased and cortical bone loss accelerated (14, 32, 33). The exact mechanisms of how increased vitamin D intake reduces circulating PTH need further investigation but are unlikely to be dependent on circulating levels of active vitamin D (3, 14, 34), although parathyroid-derived 1,25(OH)_2_D might exert intracrine effects, independent of circulating 1,25(OH)_2_D levels (3, 35, 36). Proposed mechanisms include non-1,25(OH)_2_D-mediated effects of 25OHD or other vitamin D metabolites on PTH production or stimulation via effects on vitamin D receptors in the parathyroid tissue (3, 14, 34) and/or vitamin D-mediated regulation of the expression of the calcium-sensing receptor (37, 38).

In patients with pHPT, some physicians also restrict the intake of dairy products as they are rich in both calcium and vitamin D (10). However, restriction of calcium intake has not been shown to relevantly reduce circulating calcium levels in these patients and decreases urinary calcium excretion, associated with increased bone resorption (14, 39). Otherwise, moderate supplementation with calcium (1000 mg/day) may even suppress PTH levels in these patients (21, 22). In our study, no adverse effects were observed in the subset of patients who had received combined treatment with calcium/vitamin D; however, the number of patients in this subset was very small and the dose of vitamin D provided with these preparations was insufficient in three of the five patients to increase serum 25OHD above the level of vitamin D deficiency; further studies are needed to investigate whether the use of combined calcium/vitamin D preparations is adequate and safe in patients with pHPT and coexistent vitamin D deficiency.

The strength of our study is the long-term observation period of up to 54 months in patients treated with vitamin D preparations that are commonly used in clinical practice and information about a group of patients that remained vitamin D deficient during a prolonged observation period, even though this information was collected retrospectively. Limitations include the relatively small number of cases, the observational nature of our study and, related to this, the variation in methodology; the fact that parameters that are not routinely measured in our tertiary centre such as active vitamin D 1-25(OH2)D levels and urinary calcium excretion were available in some patients only. However, in patients with pHPT, levels of 1,25(OH)_2_D are not related to those of 25OHD in cross-sectional studies (14, 15), and treatment of vitamin D-deficient patients with colecalciferol results in a strong association of 25OHD, but not of 1,25(OH)_2_D concentrations with circulating PTH (3). Furthermore, administration of active vitamin D metabolites to patients with pHPT does not decrease PTH levels (40). Although no clinical adverse effects such as symptomatic urolithiasis or deterioration of kidney function tests were observed in any of the vitamin D-treated patients in this study, larger long-term trials with matched measurement of urine calcium excretion are needed to rule out potential adverse effects on this parameter.

In conclusion, long-term replacement of vitamin D deficiency with vitamin D in various commonly prescribed preparations effectively reduced circulating PTH levels. This approach may be helpful in patients with mild pHPT and in patients who are not willing to undergo surgery or have medical contraindications. There is evidence of long-term stable biochemistry in patients with pHPT and adjCa levels up to 3 mmol/l (18), and medical treatment has been shown to be at least as effective in these patients (41), without the potential risks of a surgical intervention. By reducing circulating PTH with long-term vitamin D treatment, further stabilisation may be achieved, with potential beneficial effects on bone mass (27), although confirmation in larger studies and ideally in long-term randomised controlled studies is necessary. In the meantime, regular monitoring of adjCa and 25OHD levels is advised.

## Figures and Tables

**Table 1:** Baseline characteristics of the postmenopausal patients (*n*=40) with pHPT and coexistent vitamin D deficiency. Bone mass density was assessed using DEXA (dual energy x-ray absorptiometry) and available in 39 of the patients. Differences between groups were compared using one-way ANOVA. Data are expressed as mean±s.e.m. To convert serum 25OHD values to nanograms per millilitre, divide by 2.5.

	**Treatment group**	**Non-responders/controls**	***P* value**
*n*	28	12	
Age (years)	69±2	75±4	0.17
BMI (kg/m^2^)	29.6±1.2	29.0±2.5	0.52
25OHD (nmol/l)	32.2±1.7	33.2±2.2	0.62
PTH (pmol/l)	13.3±1.1	15.8±2.6	0.30
AdjCa (mmol/l)	2.60±0.03	2.69±0.05	0.08
ALP (U/l)	79±7	94±11	0.29
Creatinine (μmol/l)	68±2	89±7	0.001
eGFR (ml/min per 1.73 m^2^)	85±4	62±6	0.001
Use of bisphosphonates (*n*)	15/28	5/12	0.43
Osteopenia/ osteoporosis spine (*n*)	18/28	6/11	0.37
Osteopenia/ osteoporosis hip (*n*)	17/28	6/11	0.99
Observation period (month)	17±3	18±4	0.78

BMI, body mass index; pHPT, primary hyperparathyroidism; 25OHD, 25-hydroxyvitamin D; PTH, parathyroid hormone; adjCa, adjusted calcium; ALP, alkaline phosphatase; eGFR, estimated glomerular filtration rate, calculated using the modification of diet in renal disease (MDRD) formula.
